# Intravenous sildenafil acutely improves hemodynamic response to exercise in patients with connective tissue disease

**DOI:** 10.1371/journal.pone.0203947

**Published:** 2018-09-20

**Authors:** Andreas J. Rieth, Manuel J. Richter, Alexander Berkowitsch, Marc Frerix, Ingo H. Tarner, Veselin Mitrovic, Christian W. Hamm

**Affiliations:** 1 Department of Cardiology, Kerckhoff-Klinik GmbH, Bad Nauheim, Germany; 2 Department of Pneumology, Kerckhoff-Klinik GmbH, Bad Nauheim, Germany; 3 Department of Internal Medicine, Justus Liebig University Giessen, Universities of Giessen and Marburg Lung Center (UGMLC), member of the German Center for Lung Research (DZL), Giessen, Germany; 4 Department of Rheumatology and Clinical Immunology, Kerckhoff-Klinik GmbH, Bad Nauheim, Germany; 5 Department of Cardiology, Justus Liebig University Giessen, Universities of Giessen and Marburg, Giessen, Germany; Medizinische Universitat Graz, AUSTRIA

## Abstract

**Background:**

Hemodynamic assessment during exercise may unmask an impaired functional reserve of the right ventricle and the pulmonary vasculature in patients with connective tissue disease. We assessed the effect of intravenous sildenafil on the hemodynamic response to exercise in patients with connective tissue disease.

**Methods:**

In this proof-of-concept study, patients with connective tissue disease and mean pulmonary arterial pressure (mPAP) >20 mm Hg were subjected to a supine exercise hemodynamic evaluation before and after administration of intravenous sildenafil 10 mg.

**Results:**

Ten patients (four with moderately elevated mPAP 21–24 mm Hg; six with mPAP >25 mm Hg) underwent hemodynamic assessment. All of them showed markedly abnormal exercise hemodynamics. Intravenous sildenafil was well tolerated and had significant hemodynamic effects at rest and during exercise, although without pulmonary selectivity. Sildenafil reduced median total pulmonary resistance during exercise from 6.22 (IQR 4.61–8.54) to 5.24 (3.95–6.96) mm Hg·min·L^-1^ (*p* = 0.005) and increased median pulmonary arterial capacitance during exercise from 1.59 (0.93–2.28) to 1.74 (1.12–2.69) mL/mm Hg (*p* = 0.005).

**Conclusions:**

In patients with connective tissue disease who have an abnormal hemodynamic response to exercise, intravenous sildenafil improved adaption of the right ventricular-pulmonary vascular unit to exercise independent of resting mPAP. The impact of acute pharmacological interventions on exercise hemodynamics in patients with pulmonary vascular disease warrants further investigation.

**Trial registration:**

Clinicaltrials.gov NCT01889966.

## Introduction

The importance of assessing pulmonary hemodynamics during exercise, in addition to measurements made at rest, is increasingly being recognized due its diagnostic and prognostic value in different cardiopulmonary diseases [1–6]. In this context, the changes in hemodynamic parameters during exercise provide a measure of right ventricular (RV) contractile reserve and pulmonary vascular reserve [7, 8]. Hemodynamic parameters have been shown to correlate well with the results of cardiopulmonary exercise testing [6, 9].

Sildenafil, an inhibitor of phosphodiesterase 5, is frequently used to treat patients with pulmonary arterial hypertension (PAH)[10]. It leads to an elevation of cyclic guanosine monophosphate levels and thus increases signaling via the nitric oxide pathway, resulting in preferential pulmonary vasodilation[11]. An acute improvement in exercise-induced RV dysfunction by administration of sildenafil was shown in acute hypoxia [12], chronic obstructive pulmonary disease [13], chronic thromboembolic pulmonary hypertension [14], and in Fontan patients [15].

A comprehensive hemodynamic study of the acute effects of intravenous sildenafil for the assessment of the hemodynamic response to exercise in patients with connective tissue disease (CTD), who present with elevated mean pulmonary artery pressure (mPAP), has not yet been performed. We therefore conducted a proof-of-concept study to assess the RV and pulmonary vasculature responses to exercise before and after acute administration of intravenous sildenafil and to evaluate the safety of intravenous sildenafil in patients with CTD.

## Materials and methods

### Study design and setting

The present study was conducted as a prospective, open-label, single-center, single-group study between May 2013 and January 2015 at the Departments of Cardiology and Rheumatology and Clinical Immunology of the Kerckhoff-Klinik, Bad Nauheim, Germany (ClinicalTrials.gov identifier: NCT01889966). The first patient was recruited on 05/29/2013, whereas the registration at ClinicalTrials.gov was completed on 06/09/2013. This delay was due to technical problems; nevertheless the registration process was started before inclusion of the first patient. The authors confirm that all ongoing and related trials for this drug/intervention are registered. The last follow-up of the last patient was performed on 01/27/2015. The study complies with the Declaration of Helsinki, and the research protocol was approved by the locally appointed ethics committee on 11/02/2012 (University of Giessen, Approval No. 168/12). Written informed consent was obtained from every patient before any study-specific procedure was performed. The present analysis focuses on the effects of acute drug administration without taking follow-up data into account, because these have no influence on the interpretation of the acute results and were analyzed separately for reasons of clarity.

### Participants

Twenty-one patients with CTD without a previous diagnosis of PAH were screened noninvasively. They had been referred to the Department of Rheumatology and Clinical Immunology, Kerckhoff-Klinik GmbH, Bad Nauheim, Germany for inpatient treatment. Screening for PAH was performed if there was a clinical reason, e.g. worsening dyspnea or fatigue. All tests and data collection were performed in the cardiology outpatient clinic. If cardiopulmonary exercise testing (CPET), echocardiography, or N-terminal pro-brain natriuretic peptide (NT-proBNP) levels showed abnormal results, right heart catheterization was performed. Inclusion criteria were an exercise-induced rise in mPAP >30 mmHg and an abnormal pulmonary vascular reserve defined as an increase in mPAP relative to cardiac output (total pulmonary resistance, TPR) >3 mmHg/L/min. These criteria have been suggested to define exercise pulmonary hypertension [5, 6, 16].

**Fig 1:**
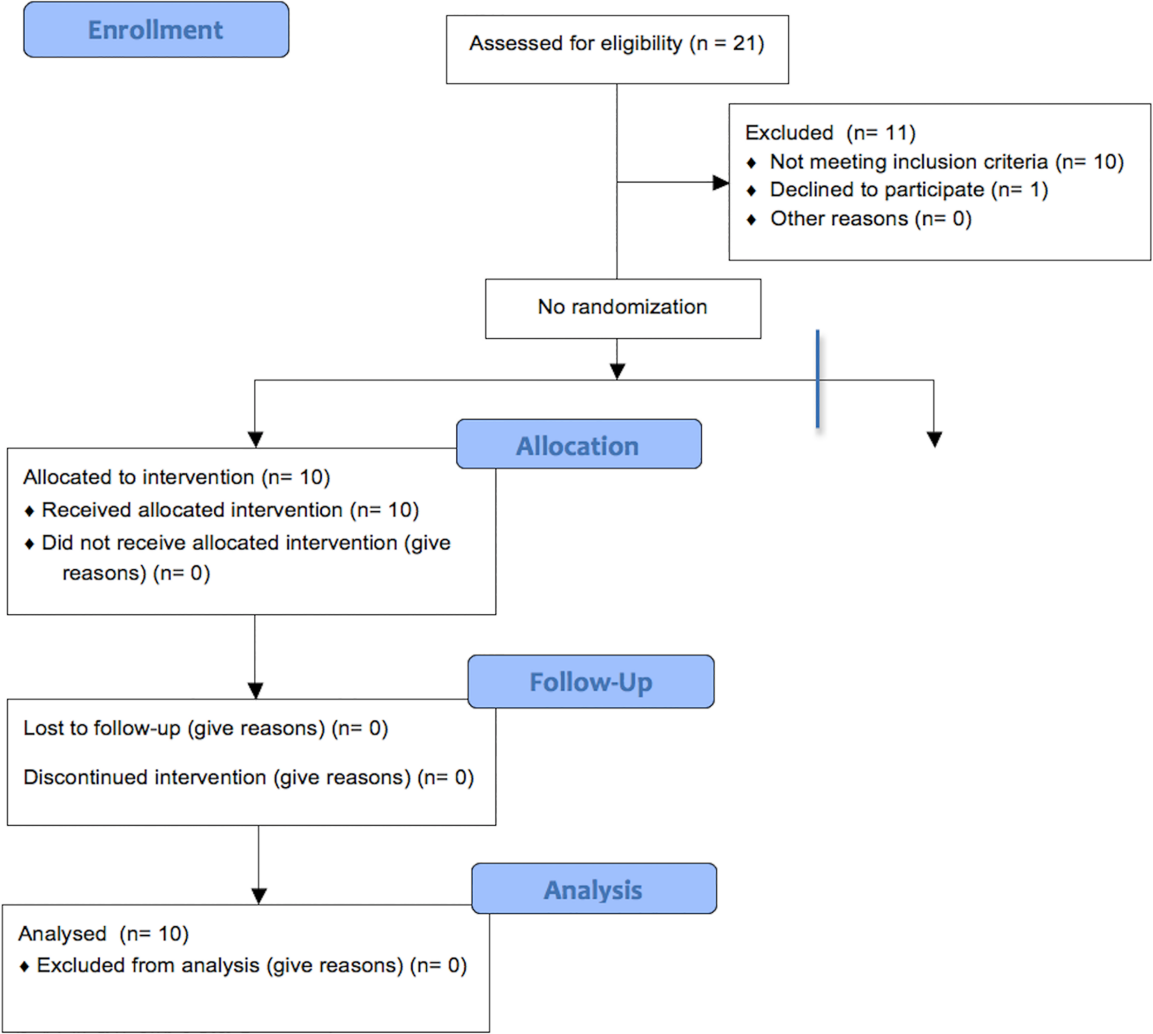
Study flow diagram.

Exclusion criteria were an inability to perform exercise tests, contraindications to sildenafil treatment, pre-treatment with PAH drugs (e.g. bosentan for digital ulcerations), pregnancy, arterial hypotension with systolic pressure repeatedly <90 mm Hg, and advanced liver or renal disease (Fig 1).

### Study treatments and assessments

The intervention was carried out in the right heart cardiac catheterization laboratory of the heart failure outpatient clinic. After baseline measurements at rest, an initial submaximal, symptom-limited exercise test using a bicycle ergometer was performed with simultaneous invasive hemodynamic measurements. After a recovery period when mPAP returned to the initially recorded value, resting patients received an intravenous injection of sildenafil 10 mg within 10 min (delivered by AJR). Comprehensive hemodynamic measurements were repeated (at rest) 15, 30, and 60 min after the start of the sildenafil injection. Finally, a second exercise test with simultaneous invasive hemodynamic measurements was performed to demonstrate the importance of these measurements [17] in showing the effect of the drug on the hemodynamic response to exercise. The individual first and second exercise tests during right heart catheterization were performed with comparable workloads and mixed-venous oxygen saturations at the end of exercise (S1 Table). All right heart catheterization and echocardiographic procedures were performed by one experienced examiner (AJR).

Hemodynamic measurements were performed in the supine position at end-expiration with a Swan-Ganz catheter (7F Thermodilution Catheter, Biosensors International, Singapore), as described previously in detail [18–20]. The pressure transducer was set at 50% of the thoracic diameter. The following parameters were measured at rest and during exercise, both before and after sildenafil injection: right atrial pressure (RAP); systolic and diastolic pulmonary arterial pressure (sPAP and dPAP, respectively); mPAP; pulmonary arterial wedge pressure (PAWP); cardiac output (CO) measured using the thermodilution method; and oxygen saturation in the pulmonary artery (SaO_2_PA). In addition, mean and systolic systemic arterial blood pressures (MAP and SAP, respectively) were measured non-invasively. Derived parameters included the following: cardiac index; RV stroke work index (RVSWI); pulmonary vascular resistance (PVR); systemic vascular resistance (SVR); ratios of PVR/SVR and mPAP/MAP (changes in these ratios following drug administration purportedly reflect pulmonary selectivity of a drug); pressure–flow relationship (mPAP/CO slope = change during exercise in mPAP [ΔmPAP]/ΔCO); total pulmonary resistance (TPR = mPAP/CO); and pulmonary arterial capacitance (PAC; stroke volume/pulse pressure = [CO/heart rate]/[sPAP−dPAP]).

### Statistical analysis

Calculations were performed using SPSS Statistics (IBM, Armonk, NY, USA). The Wilcoxon signed-rank test was used to assess the effect of intravenous sildenafil on resting and exercise hemodynamic parameters. A *p*-value <0.05 was defined as indicating a significant difference. Analyses using the Friedman test and linear mixed models were performed with standard versions implemented in SPSS V. 2.2.

## Results

### Baseline characteristics and hemodynamic response to exercise

Ten patients (seven with systemic sclerosis, two with mixed CTD, and one with systemic lupus erythematosus) were included in the study (Table 1 and S2 Table). The median age was 66.5 (interquartile range [IQR] 54.0–75.0) y, and nine of the patients were female. NYHA functional class was I−II, II, and III in two, three, and five of the patients, respectively. Echocardiographic parameters were (median [IQR]): tricuspid annular plain systolic excursion (TAPSE) 22 (14.5–25.5) mm; tricuspid annular systolic excursion velocity S´13.4 (10.9–15.0) cm/s; fractional area change (FAC) 46% (21–54); RV strain -13.85% (-9.88- -17.52); RA area 16.2 (11.8–22.3) cm^2^; tricuspid regurgitation grade 1 (0.25–1.75); RV systolic pressure 58 (48–66) mmHg. The median VÓ_2_ peak was 10.8 (8.9–13.1) mL/min/kg; breathing reserve at the end of CPET was 40 (30–47) %, with three patients ≤ 30%; V´E/VCO_2_ slope 36.5 (32.0–43.5) (S3 Table). The median 6-min walking distance (6MWD) was 270.0 (IQR 258.8–411.3) m. The results of pulmonary function testing were: forced vital capacity 60.5 (47.3–82.5) %, total lung capacity 83 (65.3–89.8) %, forced expiratory volume in one second 66.5 (50.5–75.3) %, forced expiratory volume in one second/forced vital capacity 104 (95.8–109.5)% (S4 Table). Four of the patients had moderately elevated mPAP values (21–24 mm Hg) and the remaining six had higher mPAP values (>25 mm Hg) (S5 Table).

**Table 1:** Patient characteristics.

Characteristics	Study population (n = 10)
Age, years	66.5 (54.0–75.0)
Female sex, n	9
Height, cm	168.5 (161.3–171.8)
Weight, kg	70.0 (60.3–75.8)
Connective tissue disease type, n	
Systemic sclerosis	7
Systemic lupus erythematosus	1
Mixed connective tissue disease	2
NYHA functional class, n	
I–II	2
II	3
III	5
VÓ_2_ peak, mL/min/kg	10.8 (8.9–13.1)
6MWD, m	270.0 (258.8–411.3)

Values represent median (interquartile range) unless otherwise specified. Abbreviations: NYHA, New York Heart Association; VÓ_2_ peak, peak oxygen consumption during cardiopulmonary exercise testing; 6MWD, six-minute walking distance

In the absence of sildenafil action, median mPAP increased from 27.0 (IQR 23.3–34.5) mm Hg at rest to 51.0 (IQR 47.0–56.0) mm Hg during exercise, and the exercise-induced rise in mPAP was independent of resting values. This change was accompanied by increases in median RAP, PVR, TPR, and cardiac index and a decrease in median PAC (Table 2; data for individual patients are presented in S6 Table).

**Table 2:** Acute hemodynamic effects of sildenafil at rest and during exercise.

	At rest			During exercise		
Parameter	Pre-sildenafil	30 min post-sildenafil	*p* (pre- vs. post-sildenafil)	Pre- sildenafil	Post-sildenafil	*p* (pre- vs. post-sildenafil)
sPAP, mm Hg	42.5 (37.8–53.0)	34.0 (30.3–47.3)	0.005	75.5 (67.5–93.0)	67.0 (58.3–84.5)	0.005
mPAP, mm Hg	27.0 (23.3–34.5)	22.0 (18.5–30.8)	0.005	51.0 (47.0–56.0)	44.0 (41.0–54.0)	0.007
PAWP, mm Hg	12.0 (11.0–12.8)	8.0 (6.3–10.5)	0.038	20.5 (15.5–24.0)	16.5 (11.3–21.8)	0.032
RAP, mm Hg	7.0 (6.0–8.0)	5.0 (3.3–7.8)	0.017	12.5 (9.8–21.0)	9.5 (6.3–11.0)	0.005
SAP, mm Hg	133.5 (127.5–149.8)	121.5 (111.3–128.5)	0.005	172.5 (154.0–182.8)	163.5 (152.0–180.5)	0.022
Heart rate, bpm	72.5 (62.5–75.0)	73.5 (69.3–76.5)	0.385	107.0 (98.0–112.0)	105.0 (97.8–113.0)	0.153
Cardiac index, L/min/m^2^	2.6 (2.3–2.8)	2.8 (2.6–3.2)	0.012	4.2 (3.4–5.7)	4.3 (3.8–5.6)	0.333
mPAP/CO slope, mm Hg·min·L^-1^				8.0 (5.53–10.38)	5.94 (4.85–10.92)	0.051
PVR, dyn·s·cm^−5^	278.6 (182.5–348.4)	206.6 (146.1–265.8)	0.005	327.6 (207.9–407.0)	304.3 (160.1–346.1)	0.009
SVR, dyn·s·cm^−5^	1625.9 (1119.9–1800.0)	1338.4 (912.4–1557.0)	0.007	1056.3 (804.4–1528.6)	1075.0 (812.4–1307.7)	0.575
PVR/SVR	0.2 (0.1–0.2)	0.2 (0.1–0.2)	0.171	0.3 (0.2–0.4)	0.2 (0.2–0.4)	0.102
mPAP/MAP	0.3 (0.3–0.4)	0.3 (0.2–0.4)	0.107	0.5 (0.4–0.5)	0.4 (0.3–0.5)	0.185
PAC, mL·mm Hg^−1^	2.3 (1.6–3.5)	2.5 (2.3–3.9)	0.013	1.6 (0.9–2.3)	1.7 (1.1–2.7)	0.005
TPR, mm Hg·min·L^−1^	5.7 (4.7–6.9)	4.7 (3.1–5.6)	0.005	6.2 (4.6–8.5)	5.2 (4.0–7.0)	0.005
SaO_2_PA, %	70.1 (63.5–74.4)	70.3 (61.5–76.0)	0.953	48.9 (40.3–57.0)	48.4 (40.8–61.2)	0.799
RVSWI, g·m/m^2^	11.8 (7.9–16.8)	11.4 (7.9–13.9)	0.114	20.0 (12.5–26.0)	22.0 (15.9–26.4)	0.646

Values represent median (interquartile range). MAP, mean systemic arterial pressure; mPAP, mean pulmonary arterial pressure; PAC, pulmonary arterial capacitance; PAWP, pulmonary arterial wedge pressure; PVR, pulmonary vascular resistance; RAP, right atrial pressure; RVSWI, right ventricular stroke work index; SaO_2_PA, oxygen saturation in the pulmonary artery; SAP, systolic systemic arterial pressure; sPAP, systolic pulmonary arterial pressure; SVR, systemic vascular resistance; TPR, total pulmonary resistance.

### Acute effects of intravenous sildenafil on resting and exercise hemodynamics

**Fig 2:**
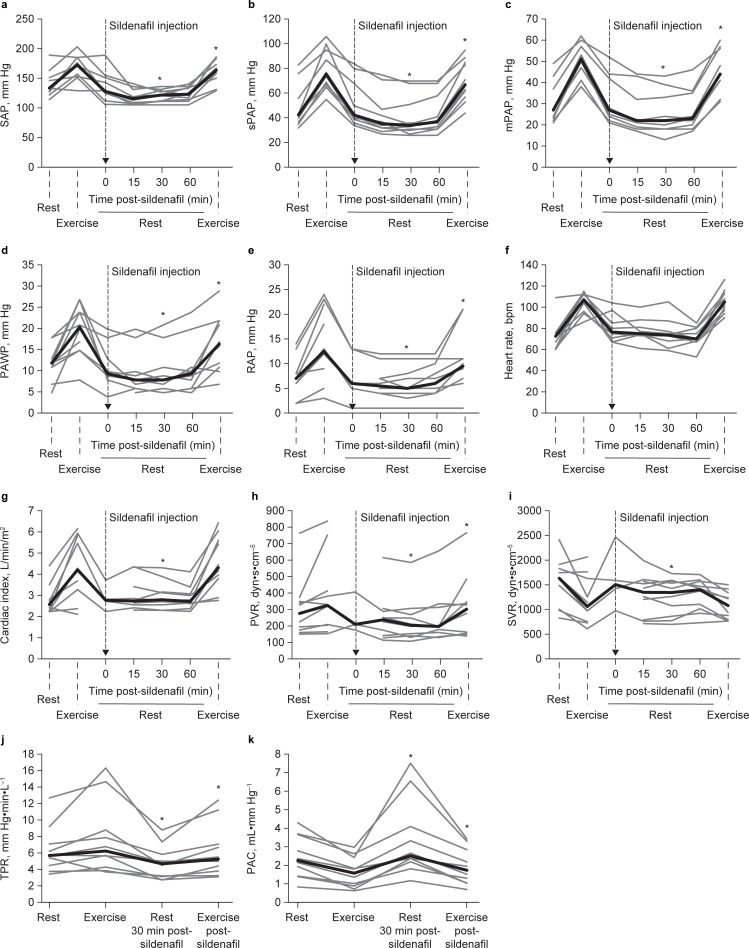
Acute responses of various parameters to intravenous sildenafil at rest and during exercise challenge. SAP (a), sPAP (b), mPAP (c), PAWP (d), RAP (e), heart rate (f), cardiac index (g), PVR (h), SVR (i), TPR (j) and PAC (k). Thin gray lines indicate individual patient data, and thick black lines indicate the median. *p < 0.05 (30 min post-sildenafil at rest vs pre-sildenafil at rest; post-sildenafil exercise challenge vs pre-sildenafil exercise challenge). mPAP, mean pulmonary arterial pressure; PAC, pulmonary arterial capacitance; PAWP, pulmonary arterial wedge pressure; PVR, pulmonary vascular resistance; RAP, right atrial pressure; SAP, systemic systolic arterial pressure; sPAP, systolic pulmonary arterial pressure; SVR, systemic vascular resistance; TPR, total pulmonary resistance.

Sildenafil injection was well tolerated during right heart catheterization. SAP dropped significantly after sildenafil injection, but none of the patients showed symptomatic hypotension. The acute hemodynamic effects of sildenafil at rest were greatest 30 min after the start of the injection. Sildenafil significantly reduced sPAP, mPAP, PAWP, and RAP as well as SAP at rest and during exercise, and it significantly increased the cardiac index at rest but not during exercise. Sildenafil also significantly reduced PVR, TPR, and PAC at rest and during exercise and SVR at rest but not during exercise. Heart rate and pulmonary-to-systemic ratios were not significantly altered by sildenafil (Fig 2 and Table 2).

The median pressure–flow relationship during exercise, expressed as the mPAP/CO slope, decreased from 8 (IQR 5.53–10.38) mm Hg/L/min without sildenafil to 5.94 (IQR 4.85–10.92) mm Hg/L/min after sildenafil injection (*p* = 0.051). Patients with mPAP <25 mmHg (N = 4) showed effects of sildenafil on PAC and TPR that were comparable to those of patients with mPAP ≥25 mmHg (N = 6), whereby exercise TPR and PAC improved more in the latter group (S7 and S8 Tables).

The results of analyses using the Friedman test and linear mixed models underline the significance of sildenafil-induced hemodynamic changes, in particular the predominant reduction of sPAP and mPAP, followed by SAP and HR. F-statistics pointed to associations between: 6MWD and HR; 6MWD and RAP; TAPSE and SVR; TAPSE and TPR; TAPSE and PVR; troponin and PAC; CAMPHOR questionnaire and CI; CAMPHOR questionnaire and the mPAP-mSAP ratio (S9 Table).

## Discussion

In the current study, we used a protocol involving exercise challenge in patients with CTD to demonstrate the following: (1) improvement of RV and pulmonary vascular functional reserve during exercise after acute administration of intravenous sildenafil; and (2) adequate safety of intravenous sildenafil administration in this setting.

We chose patients with CTD as a group with a high risk of developing PAH (e.g. estimated prevalence of 9% in systemic sclerosis)[21], which is a principal cause of death among these patients [22]. In comparison to subjects with idiopathic PAH, RV contractile reserve is more depressed in subjects with systemic sclerosis [8].

The exercise challenge demonstrated a reduced functional reserve of the RV and pulmonary vasculature independent of resting mPAP and RV function determined echocardiographically at rest, which was preserved in the majority of our patients. Mean PAP was >20 mmHg in all patients; several studies indicate that in patients with CTD, mPAP >20 mm Hg leads to an elevated risk of manifest PAH and a worse prognosis compared with mPAP ≤20 mm Hg [23–25]. Furthermore, patients with mPAP in the range of 21 to 24 mm Hg have a higher risk of developing progressive pulmonary vascular disease if they also have an exaggerated PAP response to exercise [1, 26].

In addition to the PAP response, the relationship of mPAP to CO during exercise (mPAP/CO slope, maximum TPR during exercise) is being used increasingly to detect abnormal pulmonary hemodynamics [5, 6, 27]. PAC is a relatively new parameter that reflects pulmonary vasculopathy, which is known to be associated with increased mortality in patients with PAH [28–30] and which has rarely been investigated during exercise. Both resting and exercise TPR, PVR, and PAC were markedly abnormal in our cohort. Thus, hemodynamic assessment during exercise may unmask dysfunction of the RV-pulmonary vascular circuit in CTD patients with mPAP >20 mmHg and identify patients at particularly high risk of developing PAH [31–33].

Sildenafil 10 mg administered as an intravenous injection was safe and well tolerated in these patients; neither symptomatic arterial hypotension nor other adverse reactions occurred. Although sildenafil is considered to be a highly selective inhibitor of phosphodiesterase 5 localized in the pulmonary arteries and the heart [34], sildenafil injection led to a significant decline in systemic blood pressure, SVR, and PAWP but not in ratios of pulmonary/systemic pressures and vascular resistance. Similar effects have been described in patients with left heart failure [35]. The significant reduction of PAWP following sildenafil points to an acute reduction of left ventricular (LV) afterload by inhibition of phosphodiesterase 5 in the systemic vasculature [36–38] and may also indicate an improvement of LV diastolic function [39]. Due to the serial function of the two circulations, these effects on the LV-systemic vasculature unit may have contributed to the effects on pulmonary circulation described above.

After administration of intravenous sildenafil, the second exercise challenge resulted in a different pulmonary hemodynamic response with lower maximal mPAP, PVR, and TPR, a more shallow mPAP/CO slope, and increases in PAC and CO. Measures of RV steady (PVR) and pulsatile (PAC) afterload [40], pulmonary vascular reserve (TPR) [41], and RV backward failure (RAP) showed improvement due to an acute unloading. However, aside from RAP, any of these calculated parameters depend on CO and therefore cannot be interpreted in the absence of RV contractility. Pressure-volume measurements can be used to separate RV contractility from afterload within the complex RV-pulmonary circulation unit [42, 43], but these were not performed in our study.

The sildenafil effects were mostly independent of resting mPAP. In patients with chronic thromboembolic pulmonary hypertension or chronic obstructive pulmonary disease in whom a similar approach has been applied [13, 14], exercise hemodynamic parameters of RV afterload improved after sildenafil administration to a similar extent compared with results for our cohort of CTD patients. Therefore, the results of an exercise challenge before and after administration of sildenafil appear to reflect improved RV adaptation to exercise in several different forms of pulmonary vascular disease.

It is important to emphasize that sildenafil is currently not approved for patients with resting PAWP >15 mmHg and its use in these patients is discouraged by the guidelines [11]. Therefore, apart from clinical studies, it should not be used in this population.

### Limitations

Limitations of the study are the lack of a control group and the small sample size. Another source of bias may be the multifactorial exercise limitation in CTD patients, which may be caused by pulmonary vasculopathy, respiratory limitation, and LV dysfunction (LVD) [44]. Pulmonary function tests indicated moderate restrictive pulmonary disease in most patients in our cohort. CPET revealed signs of respiratory as well as pulmonary vascular and cardiac exercise limitation. The primary focus of this study, however, was the pulmonary vascular and cardiac exercise limitation, which is the specific subject of hemodynamic measurements repeated under the same conditions including respiratory exercise limitation. Therefore, a bias of the results of hemodynamic measurements by respiratory disease seems unlikely.

We included two patients with slightly elevated resting PAWP and several patients with a significant rise in PAWP during exercise, which is indicative of coincident diastolic LVD in most of our patients. Diastolic LVD may be associated with PAH in patients with CTD [1], and it is frequently found in an elderly population [45]; as the median age was 66.5 years in our sample, the likelihood of finding PAH without diastolic LVD was low. An overlap between PAH and LVD has been described as a pathophysiological continuum [46] with a significant heterogeneity, also in clinical studies [47]. In our cohort, our observation of an acute improvement of RV and pulmonary vascular functional reserve after sildenafil administration does not seem to be affected by this heterogeneity. The exercise-associated rise in PAWP was attenuated in most of our patients after sildenafil administration (Fig 2D), so the overall sildenafil effect may result in part from an improvement of LVD.

Finally, the choice of exercise protocol may also have influenced the results. The protocol used in the current study is well established [20], however, and the observed heart rate response (Fig 2F) demonstrated reproducibility.

### Conclusions

The current proof-of-concept study provides the first evidence that intravenous sildenafil substantially enhances the hemodynamic response to exercise in patients with CTD and mPAP >20 mmHg. Our results suggest that acute pre-treatment with intravenous sildenafil is well tolerated and may improve the RV and pulmonary vascular reserve during exercise in patients with CTD independent of the degree to which resting mPAP surpasses 20 mmHg. The role of exercise hemodynamics combined with acute pharmacological interventions in pulmonary vascular diseases should be further examined. Furthermore, the investigation of PAH therapy in CTD patients with mPAP >20 mmHg might yield promising results.

## Supporting information

S1 Table Workloads and mixed-venous oxygen saturations at the end of exercise in individual patients. (DOCX)

S2 Table Characteristics of individual patients. (DOCX)

S3 Table Cardiopulmonary exercise testing in individual patients. (DOCX)

S4 Table Pulmonary function tests in individual patients. (DOCX)

S5 Table Baseline resting hemodynamics in individual patients. (DOCX)

S6 Table Baseline exercise hemodynamics in individual patients. (DOCX)

S7 Table Resting and exercise mPAP / TPR in patients with resting mPAP </≥ 25 mmHg. (DOCX)

S8 Table Resting and exercise mPAP / PAC in patients with resting mPAP </≥ 25 mmHg. (DOCX)

S9 Table Linear mixed models (H- and F-statistics). (DOCX)

S1 Document Study protocol. (PDF)

S2 Document TREND checklist. (PDF)

S3 Document CONSORT flow diagram. (DOC)

## References

[1] Lewis GD, Bossone E, Naeije R, Grunig E, Saggar R, Lancellotti P, et al Pulmonary vascular hemodynamic response to exercise in cardiopulmonary diseases. Circulation. 2013;128(13):1470–9. 10.1161/CIRCULATIONAHA.112.000667 . 24060943

[2] Chaouat A, Sitbon O, Mercy M, Poncot-Mongars R, Provencher S, Guillaumot A, et al Prognostic value of exercise pulmonary haemodynamics in pulmonary arterial hypertension. The European respiratory journal. 2014;44(3):704–13. 10.1183/09031936.00153613 . 24833765

[3] Claessen G, La Gerche A. Stressing the right ventricular-pulmonary vascular unit: beyond pulmonary vascular resistance. Heart. 2017;103(6):404–6. 10.1136/heartjnl-2016-310360 . 27729387

[4] Hasler ED, Muller-Mottet S, Furian M, Saxer S, Huber LC, Maggiorini M, et al Pressure-Flow During Exercise Catheterization Predicts Survival in Pulmonary Hypertension. Chest. 2016;150(1):57–67. 10.1016/j.chest.2016.02.634 . 26892603

[5] Herve P, Lau EM, Sitbon O, Savale L, Montani D, Godinas L, et al Criteria for diagnosis of exercise pulmonary hypertension. The European respiratory journal. 2015;46(3):728–37. 10.1183/09031936.00021915 . 26022955

[6] Kovacs G, Herve P, Barbera JA, Chaouat A, Chemla D, Condliffe R, et al An official European Respiratory Society statement: pulmonary haemodynamics during exercise. The European respiratory journal. 2017;50(5). 10.1183/13993003.00578–2017 . 29167297

[7] Spruijt OA, de Man FS, Groepenhoff H, Oosterveer F, Westerhof N, Vonk-Noordegraaf A, et al The effects of exercise on right ventricular contractility and right ventricular-arterial coupling in pulmonary hypertension. American journal of respiratory and critical care medicine. 2015;191(9):1050–7. 10.1164/rccm.201412-2271OC . 25710636

[8] Hsu S, Houston BA, Tampakakis E, Bacher AC, Rhodes PS, Mathai SC, et al Right Ventricular Functional Reserve in Pulmonary Arterial Hypertension. Circulation. 2016;133(24):2413–22. 10.1161/CIRCULATIONAHA.116.022082 ; PubMed Central PMCID: PMCPMC4907868. 27169739 PMC4907868

[9] Oliveira RKF, Faria-Urbina M, Maron BA, Santos M, Waxman AB, Systrom DM. Functional impact of exercise pulmonary hypertension in patients with borderline resting pulmonary arterial pressure. Pulmonary circulation. 2017;7(3):654–65. 10.1177/2045893217709025 . 28895507 PMC5841910

[10] Copher R, Cerulli A, Watkins A, Laura Monsalvo M. Treatment patterns and healthcare system burden of managed care patients with suspected pulmonary arterial hypertension in the United States. J Med Econ. 2012;15(5):947–55. 10.3111/13696998.2012.690801 . 22554140

[11] Galie N, Humbert M, Vachiery JL, Gibbs S, Lang I, Torbicki A, et al 2015 ESC/ERS Guidelines for the diagnosis and treatment of pulmonary hypertension: The Joint Task Force for the Diagnosis and Treatment of Pulmonary Hypertension of the European Society of Cardiology (ESC) and the European Respiratory Society (ERS): Endorsed by: Association for European Paediatric and Congenital Cardiology (AEPC), International Society for Heart and Lung Transplantation (ISHLT). European heart journal. 2016;37(1):67–119. 10.1093/eurheartj/ehv317 . 26320113

[12] Ricart A, Maristany J, Fort N, Leal C, Pages T, Viscor G. Effects of sildenafil on the human response to acute hypoxia and exercise. High Alt Med Biol. 2005;6(1):43–9. 10.1089/ham.2005.6.43 . 15772499

[13] Holverda S, Rietema H, Bogaard HJ, Westerhof N, Postmus PE, Boonstra A, et al Acute effects of sildenafil on exercise pulmonary hemodynamics and capacity in patients with COPD. Pulm Pharmacol Ther. 2008;21(3):558–64. 10.1016/j.pupt.2008.01.012 . 18342559

[14] Claessen G, La Gerche A, Wielandts JY, Bogaert J, Van Cleemput J, Wuyts W, et al Exercise pathophysiology and sildenafil effects in chronic thromboembolic pulmonary hypertension. Heart. 2015;101(8):637–44. 10.1136/heartjnl-2014-306851 . 25686630

[15] Van De Bruaene A, La Gerche A, Claessen G, De Meester P, Devroe S, Gillijns H, et al Sildenafil improves exercise hemodynamics in Fontan patients. Circulation Cardiovascular imaging. 2014;7(2):265–73. 10.1161/CIRCIMAGING.113.001243 . 24478333

[16] Godinas L, Lau EM, Chemla D, Lador F, Savale L, Montani D, et al Diagnostic concordance of different criteria for exercise pulmonary hypertension in subjects with normal resting pulmonary artery pressure. The European respiratory journal. 2016;48(1):254–7. 10.1183/13993003.01678-2015 . 27030680

[17] Chatterjee NA, Lewis GD. Characterization of pulmonary hypertension in heart failure using the diastolic pressure gradient: limitations of a solitary measurement. JACC Heart failure. 2015;3(1):17–21. 10.1016/j.jchf.2014.09.002 . 25453537

[18] Berry NC, Manyoo A, Oldham WM, Stephens TE, Goldstein RH, Waxman AB, et al Protocol for exercise hemodynamic assessment: performing an invasive cardiopulmonary exercise test in clinical practice. Pulmonary circulation. 2015;5(4):610–8. 10.1086/683815 ; PubMed Central PMCID: PMCPMC4671735. 26697168 PMC4671735

[19] Maron BA, Cockrill BA, Waxman AB, Systrom DM. The invasive cardiopulmonary exercise test. Circulation. 2013;127(10):1157–64. 10.1161/CIRCULATIONAHA.112.104463 . 23479667

[20] Rieth A, Richter MJ, Gall H, Seeger W, Ghofrani HA, Mitrovic V, et al Hemodynamic phenotyping based on exercise catheterization predicts outcome in patients with heart failure and reduced ejection fraction. The Journal of heart and lung transplantation: the official publication of the International Society for Heart Transplantation. 2017 10.1016/j.healun.2017.02.022 . 28342708

[21] Avouac J, Huscher D, Furst DE, Opitz CF, Distler O, Allanore Y, et al Expert consensus for performing right heart catheterisation for suspected pulmonary arterial hypertension in systemic sclerosis: a Delphi consensus study with cluster analysis. Annals of the rheumatic diseases. 2014;73(1):191–7. 10.1136/annrheumdis-2012-202567 . 23349131

[22] Mathai SC, Hassoun PM. Pulmonary arterial hypertension associated with systemic sclerosis. Expert Rev Respir Med. 2011;5(2):267–79. 10.1586/ers.11.18 ; PubMed Central PMCID: PMCPMC3100897. 21510736 PMC3100897

[23] Bae S, Saggar R, Bolster MB, Chung L, Csuka ME, Derk C, et al Baseline characteristics and follow-up in patients with normal haemodynamics versus borderline mean pulmonary arterial pressure in systemic sclerosis: results from the PHAROS registry. Annals of the rheumatic diseases. 2012;71(8):1335–42. 10.1136/annrheumdis-2011-200546 ; PubMed Central PMCID: PMCPMC3398226. 22307943 PMC3398226

[24] Heresi GA, Minai OA, Tonelli AR, Hammel JP, Farha S, Parambil JG, et al Clinical characterization and survival of patients with borderline elevation in pulmonary artery pressure. Pulmonary circulation. 2013;3(4):916–25. 10.1086/674756 ; PubMed Central PMCID: PMCPMC4070822. 25006408 PMC4070822

[25] Suzuki A, Taniguchi H, Watanabe N, Kondoh Y, Kimura T, Kataoka K, et al Significance of pulmonary arterial pressure as a prognostic indicator in lung-dominant connective tissue disease. PloS one. 2014;9(9):e108339 10.1371/journal.pone.0108339 ; PubMed Central PMCID: PMCPMC4182458. 25268705 PMC4182458

[26] Stamm A, Saxer S, Lichtblau M, Hasler ED, Jordan S, Huber LC, et al Exercise pulmonary haemodynamics predict outcome in patients with systemic sclerosis. The European respiratory journal. 2016;48(6):1658–67. 10.1183/13993003.00990-2016 . 27824602

[27] Naeije R, Vanderpool R, Dhakal BP, Saggar R, Saggar R, Vachiery JL, et al Exercise-induced pulmonary hypertension: physiological basis and methodological concerns. American journal of respiratory and critical care medicine. 2013;187(6):576–83. 10.1164/rccm.201211-2090CI ; PubMed Central PMCID: PMC3733438. 23348976 PMC3733438

[28] Dragu R, Rispler S, Habib M, Sholy H, Hammerman H, Galie N, et al Pulmonary arterial capacitance in patients with heart failure and reactive pulmonary hypertension. European journal of heart failure. 2015;17(1):74–80. 10.1002/ejhf.192 . 25388783

[29] Tan W, Madhavan K, Hunter KS, Park D, Stenmark KR. Vascular stiffening in pulmonary hypertension: cause or consequence? (2013 Grover Conference series). Pulmonary circulation. 2014;4(4):560–80. 10.1086/677370 ; PubMed Central PMCID: PMC4278618. 25610594 PMC4278618

[30] Wang Z, Chesler NC. Pulmonary vascular wall stiffness: An important contributor to the increased right ventricular afterload with pulmonary hypertension. Pulmonary circulation. 2011;1(2):212–23. 10.4103/2045-8932.83453 ; PubMed Central PMCID: PMCPMC3198648. 22034607 PMC3198648

[31] Steen V, Chou M, Shanmugam V, Mathias M, Kuru T, Morrissey R. Exercise-induced pulmonary arterial hypertension in patients with systemic sclerosis. Chest. 2008;134(1):146–51. 10.1378/chest.07-2324 . 18403670

[32] Park MH, Ramani GV, Kop WJ, Uber PA, Mehra MR. Exercise-uncovered pulmonary arterial hypertension and pharmacologic therapy: Clinical benefits. The Journal of heart and lung transplantation: the official publication of the International Society for Heart Transplantation. 2010;29(2):228–9. 10.1016/j.healun.2009.10.010 . 20113912

[33] Chia EM, Lau EM, Xuan W, Celermajer DS, Thomas L. Exercise testing can unmask right ventricular dysfunction in systemic sclerosis patients with normal resting pulmonary artery pressure. International journal of cardiology. 2016;204:179–86. 10.1016/j.ijcard.2015.11.186 . 26681539

[34] Nagendran J, Archer SL, Soliman D, Gurtu V, Moudgil R, Haromy A, et al Phosphodiesterase type 5 is highly expressed in the hypertrophied human right ventricle, and acute inhibition of phosphodiesterase type 5 improves contractility. Circulation. 2007;116(3):238–48. 10.1161/CIRCULATIONAHA.106.655266 . 17606845

[35] Botha P, Parry G, Dark JH, Macgowan GA. Acute hemodynamic effects of intravenous sildenafil citrate in congestive heart failure: comparison of phosphodiesterase type-3 and -5 inhibition. The Journal of heart and lung transplantation: the official publication of the International Society for Heart Transplantation. 2009;28(7):676–82. 10.1016/j.healun.2009.04.013 . 19560695

[36] Archer SL, Michelakis ED. Phosphodiesterase type 5 inhibitors for pulmonary arterial hypertension. The New England journal of medicine. 2009;361(19):1864–71. 10.1056/NEJMct0904473 . 19890129

[37] Lin CS. Tissue expression, distribution, and regulation of PDE5. Int J Impot Res. 2004;16 Suppl 1:S8–S10. 10.1038/sj.ijir.3901207 . 15224128

[38] Lin CS, Lin G, Xin ZC, Lue TF. Expression, distribution and regulation of phosphodiesterase 5. Curr Pharm Des. 2006;12(27):3439–57. . 17017938 10.2174/138161206778343064

[39] Bishu K, Hamdani N, Mohammed SF, Kruger M, Ohtani T, Ogut O, et al Sildenafil and B-type natriuretic peptide acutely phosphorylate titin and improve diastolic distensibility in vivo. Circulation. 2011;124(25):2882–91. 10.1161/CIRCULATIONAHA.111.048520 ; PubMed Central PMCID: PMC3412357. 22144574 PMC3412357

[40] Rosenkranz S, Hoeper M. Pulmonary vascular indices and survival in left heart disease: illusion of conclusion? European journal of heart failure. 2017 10.1002/ejhf.1022 10.1002/ejhf.860 29052305

[41] Claessen G, La Gerche A, Petit T, Gillijns H, Bogaert J, Claeys M, et al Right ventricular and pulmonary vascular reserve in asymptomatic BMPR2 mutation carriers. The Journal of heart and lung transplantation: the official publication of the International Society for Heart Transplantation. 2017;36(2):148–56. 10.1016/j.healun.2016.06.018 . 27475894

[42] Vonk Noordegraaf A, Westerhof BE, Westerhof N. The Relationship Between the Right Ventricle and its Load in Pulmonary Hypertension. Journal of the American College of Cardiology. 2017;69(2):236–43. 10.1016/j.jacc.2016.10.047 . 28081831

[43] Naeije R, Brimioulle S, Dewachter L. Biomechanics of the right ventricle in health and disease (2013 Grover Conference series). Pulmonary circulation. 2014;4(3):395–406. 10.1086/677354 ; PubMed Central PMCID: PMC4278599. 25621153 PMC4278599

[44] Boutou AK, Pitsiou GG, Siakka P, Dimitroulas T, Paspala A, Sourla E, et al Phenotyping Exercise Limitation in Systemic Sclerosis: The Use of Cardiopulmonary Exercise Testing. Respiration. 2016;91(2):115–23. 10.1159/000442888 . 26731293

[45] Ponikowski P, Voors AA, Anker SD, Bueno H, Cleland JG, Coats AJ, et al 2016 ESC Guidelines for the diagnosis and treatment of acute and chronic heart failure: The Task Force for the diagnosis and treatment of acute and chronic heart failure of the European Society of Cardiology (ESC)Developed with the special contribution of the Heart Failure Association (HFA) of the ESC. European heart journal. 2016;37(27):2129–200. 10.1093/eurheartj/ehw128 . 27206819

[46] Opitz CF, Hoeper MM, Gibbs JS, Kaemmerer H, Pepke-Zaba J, Coghlan JG, et al Pre-Capillary, Combined, and Post-Capillary Pulmonary Hypertension: A Pathophysiological Continuum. Journal of the American College of Cardiology. 2016;68(4):368–78. 10.1016/j.jacc.2016.05.047 . 27443433

[47] Hoeper M, Galie N, Barbera JA, Frost A, Ghofrani A, McLaughlin V, et al Initial combination therapy with ambrisentan (AMB) and tadalafil (TAD) in treatment naïve patients with pulmonary arterial hypertension (PAH): Efficacy and safety in the AMBITION study intent to treat (ITT) population. European Respiratory Journal. 2015;46(suppl 59). 10.1183/13993003.congress-2015.OA4994

