# Supplementary material for: Intravenous sildenafil acutely improves hemodynamic response to exercise in patients with connective tissue disease
Source: PLoS One. 2018 Sep 20;13(9):e0203947. doi: 10.1371/journal.pone.0203947 (PMC6147445; doi:10.1371/journal.pone.0203947)
Supplement: S1 Table — (DOCX) [file pone.0203947.s001.docx]

**S1 Table:** **Workloads and mixed-venous oxygen saturations at the end of exercise in individual patients**

##

| **ID** | **SvO2 (rest)** | **SvO2 (exerise1)** | **SvO2 (exercise2)** | **Workload (exercise 1)** | **Workload (exercise 2)** |
| --- | --- | --- | --- | --- | --- |
| 1 | 79.5 | 65.4 | 60.0 | 25W/3 min, 10W/3 min | 25W/3 min, 10W/7 min |
| 2 | 71.1 | 48.2 | 48.6 | 50W/9 min | 50W/9 min |
| 3 | 67.5 | 51.9 | 47.2 | 25W/6 min | 25W/7 min |
| 4 | 58.4 | 32.3 | 35.6 | 25W/8 min | 25W/8 min |
| 5 | 57.5 | 39.8 | 40.3 | 5W/3,25 min | 5W/5 min |
| 6 | 79.5 | 66.4 | 65.9 | 25W/8 min | 25W/8 min |
| 7 | 69.1 | 34.7 | 35.7 | 15W/5 min | 15W/5 min |
| 8 | 62.2 | 41.6 | 41.0 | 0W/5 min | 0W/5 min |
| 9 | 75.3 | 58.7 | 64.8 | 25W/8 min | 25W/8 min |
| 10 | 71.6 | 49.5 | 49.5 | 25W/7 min | 25W/2 min, 15W/3 min |

## ID, identification number; SvO2, mixed-venous oxygen saturation; W, watts; min, minutes.
