# Supplementary material for: Intravenous sildenafil acutely improves hemodynamic response to exercise in patients with connective tissue disease
Source: PLoS One. 2018 Sep 20;13(9):e0203947. doi: 10.1371/journal.pone.0203947 (PMC6147445; doi:10.1371/journal.pone.0203947)
Supplement: S2 Table — (DOCX) [file pone.0203947.s002.docx]

## S2 Table: Characteristics of individual patients

| **ID** | **CTD type** | **Age, years** | **Height, cm** | **Weight, kg** | **Sex** | **NYHA FC** | **VÓ_2_ peak, mL/min/kg** | **6MWD, m** |
| --- | --- | --- | --- | --- | --- | --- | --- | --- |
| 1 | SSc | 72 | 158 | 72 | f | II | 11.3 | 255 |
| 2 | SLE | 52 | 172 | 61 | f | I–II | 26.3 | 630 |
| 3 | SSc | 69 | 171 | 68 | f | III | 12.2 | 400 |
| 4 | MCTD | 78 | 172 | 102 | m | II | 9.1 | 270 |
| 5 | MCTD | 60 | 169 | 60 | f | III | 6.8 | 210 |
| 6 | SSc | 46 | 168 | 96 | f | II | 8.6 | 270 |
| 7 | SSc | 76 | 158 | 72 | f | III | 9.9 | 270 |
| 8 | SSc | 76 | 165 | 60 | f | III | 9.7 | 84 |
| 9 | SSc | 27 | 180 | 60 | f | I–II | 14.0 | 556 |
| 10 | SSc | 64 | 160 | 77 | f | III | 11.7 | 415 |

ID, identification number; 6MWD, 6-minute walk distance; CTD, connective tissue disease; f, female; ID, identification number; m, male; MCTD, mixed connective tissue disease; NYHA FC, New York Heart Association functional class; SLE, systemic lupus erythematosus; SSc, systemic sclerosis; VÓ_2_ peak, peak oxygen consumption during cardiopulmonary exercise testing.
