# Supplementary material for: Intravenous sildenafil acutely improves hemodynamic response to exercise in patients with connective tissue disease
Source: PLoS One. 2018 Sep 20;13(9):e0203947. doi: 10.1371/journal.pone.0203947 (PMC6147445; doi:10.1371/journal.pone.0203947)
Supplement: S3 Table — (DOCX) [file pone.0203947.s003.docx]

**S3 Table:** **Cardiopulmonary exercise testing in individual patients**

| **ID** | **max. work-**  **load (W)** | **max. HR (/min.)** | **max. HR (%pred.)** | **V´O2 peak. (ml/min/kg)** | **V´O2 peak (%pred.)** | **RER** | **V´E/V´CO2 slope** | **O2/HR (/min.)** | **O2/HR (%pred.)** | **BR (%)** |
| --- | --- | --- | --- | --- | --- | --- | --- | --- | --- | --- |
| 1 | 40 | 133 | 90 | 11.3 | 70 | 1.01 | 32 | 6.1 | 75 | 40 |
| 2 | 100 | 166 | 94 | 26.3 | 107 | 1.08 | 30 | 9.8 | 81 | n.a. |
| 3 | 40 | 111 | 74 | 12.2 | 67 | 1.03 | 57 | 7.4 | 92 | 35 |
| 4 | 40 | 118 | 83 | 9.1 | 53 | 0.93 | 38 | 8.1 | 50 | 30 |
| 5 | 30 | 126 | 79 | 6.8 | 33 | 1.24 | 70 | 3.6 | 42 | 16 |
| 6 | 50 | 116 | 67 | 8.6 | 45 | 0.95 | 45 | 7.1 | 59 | 48 |
| 7 | 30 | 110 | 76 | 9.9 | 65 | 1.02 | 35 | 6.4 | 82 | 2 |
| 8 | 20 | 108 | 74 | 9.7 | 56 | 1.04 | 39 | 5.4 | 76 | 44 |
| 9 | 60 | 137 | 71 | 14.0 | 42 | 1.29 | 24 | 6.2 | 63 | 77 |
| 10 | 80 | 125 | 80 | 11.7 | 67 | 1.25 | 32 | 7.0 | 78 | 47 |
|  |  |  |  |  |  |  |  |  |  |  |
| Med. | 40.0 | 121.50 | 77.50 | 10.60 | 60.50 | 1.04 | 36.50 | 6.70 | 75.50 | 40 |
| IQR 25 | 32.5 | 112.25 | 74.00 | 9.25 | 47.00 | 1.01 | 32.00 | 6.13 | 60.00 | 30 |
| IQR 75 | 57.5 | 131.25 | 82.25 | 12.08 | 67.00 | 1.20 | 43.50 | 7.33 | 80.25 | 47 |

ID, identification number; HR, heart rate; pred., predicted; VÓ_2_ peak, peak oxygen consumption during cardiopulmonary exercise testing; RER, respiratory exchange ratio; V´E/V´CO2 slope, breathing equivalent of CO2; O2/HR, oxygen pulse; BR, breathing reserve.
