# Supplementary material for: Intravenous sildenafil acutely improves hemodynamic response to exercise in patients with connective tissue disease
Source: PLoS One. 2018 Sep 20;13(9):e0203947. doi: 10.1371/journal.pone.0203947 (PMC6147445; doi:10.1371/journal.pone.0203947)
Supplement: S4 Table — (DOCX) [file pone.0203947.s004.docx]

**S4 Table:** **Pulmonary function tests in individual patients**

| **ID** | **FVC (L)** | **FVC (%pred.)** | **TLC (L)** | **TLC (%pred.)** | **FEV1 (L)** | **FEV1 (%pred.)** | **FEV1%VC (%)** |
| --- | --- | --- | --- | --- | --- | --- | --- |
| 1 | 1.01 | 45 | 2.96 | 64 | 0.85 | 46 | 81 |
| 2 | 4.54 | 133 | 6.66 | 118 | 3.94 | 134 | 104 |
| 3 | 1.85 | 64 | 2.97 | 54 | 1.66 | 68 | 117 |
| 4 | 1.69 | 46 | 5.79 | 84 | 1.33 | 48 | 101 |
| 5 | 1.78 | 57 | 4.99 | 92 | 1.72 | 65 | 124 |
| 6 | 3.01 | 90 | 4.79 | 90 | 2.2 | 76 | 82 |
| 7 | 0.79 | 40 | 2.23 | 51 | 0.69 | 44 | 94 |
| 8 | 1.24 | 51 | 3.5 | 69 | 1.16 | 58 | 104 |
| 9 | 2.85 | 66 | 4.97 | 82 | 2.76 | 73 | 111 |
| 10 | 2.2 | 88 | 4.16 | 89 | 1.82 | 88 | 105 |
|  |  |  |  |  |  |  |  |
| median | 1.82 | 60.50 | 4.48 | 83.00 | 1.69 | 66.50 | 104.00 |
| IQR 25 | 1.35 | 47.25 | 3.10 | 65.25 | 1.20 | 50.50 | 95.75 |
| IQR 75 | 2.69 | 82.50 | 4.99 | 89.75 | 2.11 | 75.25 | 109.50 |

ID, identification number; FVC, forced vital capacity; L, liters; pred., predicted; TLC, total lung capacity; FEV1, forced expiratory volume in one second; FEV1%VC, forced expiratory volume in one second/forced vital capacity.
