# Supplementary material for: Intravenous sildenafil acutely improves hemodynamic response to exercise in patients with connective tissue disease
Source: PLoS One. 2018 Sep 20;13(9):e0203947. doi: 10.1371/journal.pone.0203947 (PMC6147445; doi:10.1371/journal.pone.0203947)
Supplement: S5 Table — (DOCX) [file pone.0203947.s005.docx]

**S5 Table:** **Resting hemodynamics in individual patients**

| **ID** | **mPAP, mm Hg** | **PAWP, mm Hg** | **RAP, mm Hg** | **Cardiac index, L/min/m^2^** | **PVR, dyn·s·cm^−5^** | **PAC, mL/mm Hg** | **TPR, mm Hg·min·L^−1^** |
| --- | --- | --- | --- | --- | --- | --- | --- |
| 1 | 21 | 12 | 7 | 2.7 | 153 | 2.2 | 4.5 |
| 2 | 27 | 7 | 2 | 2.4 | 340 | 2.4 | 5.7 |
| 3 | 23 | 5 | 2 | 2.3 | 351 | 1.4 | 5.6 |
| 4 | 43 | 18 | 13 | 2.8 | 330 | 1.4 | 7.1 |
| 5 | 49 | 12 | 14 | 2.2 | 765 | 0.9 | 12.7 |
| 6 | 27 | 11 | 6 | 3.5 | 178 | 4.3 | 3.8 |
| 7 | 24 | 13 | 7 | 2.2 | 228 | 2.8 | 6.2 |
| 8 | 37 | 18 | 8 | 2.4 | 377 | 1.9 | 9.2 |
| 9 | 22 | 12 | 8 | 2.3 | 197 | 3.7 | 5.4 |
| 10 | 27 | 11 | 6 | 4.4 | 163 | 3.7 | 3.4 |

ID, identification number; mPAP, mean pulmonary arterial pressure; PAC, pulmonary arterial capacitance; PAWP, pulmonary artery wedge pressure; PVR, pulmonary vascular resistance; RAP, right atrial pressure; TPR, total pulmonary resistance.
