# Supplementary material for: Intravenous sildenafil acutely improves hemodynamic response to exercise in patients with connective tissue disease
Source: PLoS One. 2018 Sep 20;13(9):e0203947. doi: 10.1371/journal.pone.0203947 (PMC6147445; doi:10.1371/journal.pone.0203947)
Supplement: S6 Table — (DOCX) [file pone.0203947.s006.docx]

## S6 Table: Exercise hemodynamics in individual patients

| **ID** | **mPAP, mm Hg** | **PAWP, mm Hg** | **RAP, mm Hg** | **Cardiac index, L/min/m^2^** | **PVR, dyn·s·cm^−5^** | **PAC, mL/mm Hg** | **TPR, mm Hg⋅min⋅L^−1^** |
| --- | --- | --- | --- | --- | --- | --- | --- |
| 1 | 41 | 27 | 12 | 4.2 | 156 | 1.4 | 5.7 |
| 2 | 53 | 8 | 3 | 5.5 | 383 | 1.8 | 5.6 |
| 3 | 52 | 20 | 5 | 4.3 | 332 | 0.9 | 6.8 |
| 4 | 62 | 21 | 23 | 3.7 | 415 | 1.0 | 7.8 |
| 5 | 60 | 17 | 24 | 2.4 | 839 | 0.6 | 14.6 |
| 6 | 47 | 15 | 12 | 6.0 | 212 | 2.4 | 3.9 |
| 7 | 50 | 27 | 18 | 3.3 | 323 | 1.8 | 8.8 |
| 8 | 57 | 24 | 22 | 2.1 | 754 | 0.7 | 16.3 |
| 9 | 38 | 15 | 9 | 5.8 | 207 | 3.0 | 3.7 |
| 10 | 47 | 24 | 13 | 6.2 | 167 | 2.6 | 4.3 |

ID, identification number; mPAP, mean pulmonary arterial pressure; PAC, pulmonary arterial capacitance; PAWP, pulmonary artery wedge pressure; PVR, pulmonary vascular resistance; RAP, right atrial pressure; TPR, total pulmonary resistance.
