# Supplementary material for: Intravenous sildenafil acutely improves hemodynamic response to exercise in patients with connective tissue disease
Source: PLoS One. 2018 Sep 20;13(9):e0203947. doi: 10.1371/journal.pone.0203947 (PMC6147445; doi:10.1371/journal.pone.0203947)
Supplement: S7 Table — (DOCX) [file pone.0203947.s007.docx]

## S7 Table: Resting and exercise mPAP / TPR in patients with resting mPAP < / ≥ 25 mmHg

| **ID** | **mPAP, mm Hg**  **(rest)** | **mPAP, mm Hg**  **(exercise)** | **TPR, mm Hg⋅min⋅L^−1^ (rest pre sildenafil)** | **TPR, mm Hg⋅min⋅L^−1^**  **(rest 30 min post-sildenafil)** | **Δ TPR, mm Hg⋅min⋅L^−1^**  **(rest)** | **TPR, mm Hg⋅min⋅L^−1^**  **(exercise pre sildenafil)** | **TPR, mm Hg⋅min⋅L^−1^**  **(exercise post-sildenafil)** | **Δ TPR, mm Hg⋅min⋅L^−1^**  **(exercise)** |
| --- | --- | --- | --- | --- | --- | --- | --- | --- |
| 1 | 21.0 | 41.0 | 4.47 | 2.71 | -1.76 | 5.69 | 4.41 | -1.28 |
| 3 | 23.0 | 52.0 | 5.61 | 4.84 | -0.77 | 6.75 | 5.50 | -1.25 |
| 7 | 24.0 | 50.0 | 6.2 | 4.50 | -1.70 | 8.77 | 6.67 | -2.11 |
| 9 | 22.0 | 38.0 | 5.41 | 3.21 | -2.19 | 3.69 | 3.23 | -0.46 |
| **mean** | **22.5** | **45.3** | **5.42** | **3.82** | **-1.60** | **6.23** | **4.95** | **-1.27** |
|  |  |  |  |  |  |  |  |  |
| 2 | 27.0 | 53.0 | 5.74 | 5.00 | -0.74 | 5.64 | 4.97 | -0.66 |
| 4 | 43.0 | 62.0 | 7.08 | 5.82 | -1.26 | 7.85 | 7.06 | -0.79 |
| 5 | 49.0 | 60.0 | 12.66 | 8.78 | -3.89 | 14.63 | 11.20 | -3.43 |
| 6 | 27.0 | 47.0 | 3.75 | 2.75 | 1.00 | 3.85 | 3.11 | -0.75 |
| 8 | 37.0 | 57.0 | 9.18 | 7.33 | -1.85 | 16.29 | 12.39 | -3.89 |
| 10 | 27.0 | 47.0 | 3.43 | 3.12 | -0.31 | 4.27 | 3.80 | -0.48 |
| **mean** | **35.0** | **54.3** | **6.98** | **5.47** | **-1.51** | **8.76** | **7.09** | **-1.67** |

ID, identification number; mPAP, mean pulmonary arterial pressure; TPR, total pulmonary resistance; **Δ,** difference between pre / post sildenafil.
