# Supplementary material for: Intravenous sildenafil acutely improves hemodynamic response to exercise in patients with connective tissue disease
Source: PLoS One. 2018 Sep 20;13(9):e0203947. doi: 10.1371/journal.pone.0203947 (PMC6147445; doi:10.1371/journal.pone.0203947)
Supplement: S8 Table — (DOCX) [file pone.0203947.s008.docx]

## S8 Table: Resting and exercise PAC in patients with resting mPAP < / ≥ 25 mmHg

| **ID** | **mPAP, mm Hg**  **(rest)** | **mPAP, mm Hg**  **(exercise)** | **PAC, mL/mm Hg**  **(rest pre sildenafil)** | **PAC, mL/mm Hg**  **(rest 30 min post-sildenafil)** | | **Δ PAC mL/mm Hg**  **(rest)** | **PAC, mL/mm Hg**  **(exercise pre sildenafil)** | **PAC, mL/mm Hg**  **(exercise post-sildenafil)** | **Δ PAC mL/mm Hg**  **(exercise)** | |  |
| --- | --- | --- | --- | --- | --- | --- | --- | --- | --- | --- | --- |
| 1 | 21.0 | 41.0 | 2.16 | 2.67 | 0.51 | | 1.37 | 1.53 | | 0.16 | |
| 3 | 23.0 | 52.0 | 1.40 | 2.20 | 0.80 | | 0.90 | 1.06 | | 0.16 | |
| 7 | 24.0 | 50.0 | 2.79 | 2.38 | -0.41 | | 1.84 | 1.96 | | 0.12 | |
| 9 | 22.0 | 38.0 | 3.71 | 6.56 | 2.85 | | 2.99 | 3.31 | | 0.32 | |
| **mean** | **22.5** | **45.3** | **2.51** | **3.45** | **0.94** | | **1.77** | **1.96** | | **0.19** | |
|  |  |  |  |  |  | |  |  | |  | |
| 2 | 27.0 | 53.0 | 2.37 | 3.33 | 0.96 | | 1.81 | 2.19 | | 0.39 | |
| 4 | 43.0 | 62.0 | 1.43 | 1.82 | 0.40 | | 1.03 | 1.31 | | 0.28 | |
| 5 | 49.0 | 60.0 | 0.85 | 1.18 | 0.34 | | 0.64 | 0.71 | | 0.07 | |
| 6 | 27.0 | 47.0 | 4.31 | 7.52 | 3.21 | | 2.43 | 3.44 | | 1.00 | |
| 8 | 37.0 | 57.0 | 1.94 | 2.38 | 0.44 | | 0.73 | 1.04 | | 0.31 | |
| 10 | 27.0 | 47.0 | 3.67 | 4.10 | 0.43 | | 2.64 | 2.85 | | 0.22 | |
| **mean** | **35.0** | **54.3** | **2.43** | **3.39** | **0.96** | | **1.55** | **1.92** | | **0.38** | |

ID, identification number; mPAP, mean pulmonary arterial pressure; PAC, pulmonary arterial capacitance; **Δ,** difference between pre / post sildenafil.
