# Supplementary material for: Intravenous sildenafil acutely improves hemodynamic response to exercise in patients with connective tissue disease
Source: PLoS One. 2018 Sep 20;13(9):e0203947. doi: 10.1371/journal.pone.0203947 (PMC6147445; doi:10.1371/journal.pone.0203947)
Supplement: S9 Table — (DOCX) [file pone.0203947.s009.docx]

## S9 Table: Linear mixed models (H- and F-statistics)

|  | **H-Statistics** | **p** |  |  | **Fixed effects (Impact of all factors are**  **given in F-Statistics).** | |  |  |
| --- | --- | --- | --- | --- | --- | --- | --- | --- |
|  |  |  | **Time Point** | **6MWD** | **TAPSE** | **Troponin** | | **Camphor** |
| **PAWP** | 32.575 | <.001 | 8.505 | 12.499 | 12.510 | 1.685 | | 0.830 |
| **sPAP** | 54.532 | <.001 | 41.795 | 3.403 | 0.163 | 0.141 | | 0.213 |
| **mPAP** | 56.38 | <.001 | 81.451 | 0.112 | 26.328 | 0.579 | | 0.392 |
| **PAC** | 28.92 | <.001 | 10.316 | 4.265 | 18.098 | 10.582 | | 0.525 |
| **TPR** | 23.4 | <.001 | 6.786 | 2.171 | 34.744 | 3.161 | | 0.985 |
| **RAP** | 36.805 | <.001 | 10.591 | 21.750 | 6.060 | 5.192 | | 0.328 |
| **SAP** | 48.61 | <.001 | 23.449 | 16.075 | 2.799 | 5.219 | | 0.002 |
| **HR** | 42.768 | <.001 | 34.592 | 36.925 | 7.342 | 0.103 | | 5.390 |
| **CI** | 29.67 | <.001 | 12.843 | 18.088 | 1.599 | 0.525 | | 21.190 |
| **PVR** | 26.114 | <.001 | 3.537 | 16.759 | 30.655 | 1.453 | | 0.865 |
| **SVR** | 17.178 | 0.004 | 7.503 | 9.908 | 73.929 | 0.079 | | 3.942 |
| **RP/RS** | 23.9 | <.001 | 7.831 | 5.749 | 0.254 | 0.003 | | 0.007 |
| **mPAP/mSAP** | 35.83 | <.001 | 19.218 | 1.581 | 0.032 | 0.053 | | 20.646 |
| **SvO2** | 19.481 | <.001 | 32.017 | 9.395 | 23.402 | 0.499 | | 4.989 |

PAWP, pulmonary artery wedge pressure; sPAP, systolic pulmonary arterial pressure; mPAP, mean pulmonary arterial pressure; PAC, pulmonary arterial capacitance; TPR, total pulmonary resistance; RAP, right atrial pressure; SAP, systemic arterial pressure; HR, heart rate; CI, cardiac index; PVR, pulmonary vascular resistance; RP/RS, PVR/systemic vascular resistance, mPAP/mSAP, mPAP/ mean SAP; SvO2, mixed-venous oxygen saturation; 6MWD, 6-minute walk distance; TAPSE, tricuspid annular plain systolic excursion.
