# Supplementary material for: Intravenous sildenafil acutely improves hemodynamic response to exercise in patients with connective tissue disease
Source: PLoS One. 2018 Sep 20;13(9):e0203947. doi: 10.1371/journal.pone.0203947 (PMC6147445; doi:10.1371/journal.pone.0203947)
Supplement: S1 Document — (PDF) [file pone.0203947.s010.pdf]

SIL-REACT

Vaso**REACT**ivity Testing with Intravenous **SIL**denafil

in Patients with Precapillary Pulmonary Hypertension

Investigator:

- Dr. A. Rieth

  Kerckhoff-Klinik Center for Heart-, Lung- and Rheumatology Diseases

Co-Investigators:

- Dr. W. Hermann
- Prof. Dr. V. Mitrovic
- Prof. Dr. C. Hamm

Address for correpondence:

Dr. Andreas Rieth

Kerckhoff-Klinik Center for Heart-, Lung- and

Rheumatology Testing

Dept. of Cardiology

Benekestraße 2-8

61231 Bad Nauheim, Germany

Tel.: +49 6032 996-0 (switch); Fax: +49 6032 996-2681

email: a.rieth@kerckhoff-klinik.de

Background

The so-called vasoreactivity testing is an inherent part of invasive diagnostics of pulmonary hypertension (PH) [1]. It has to be clarified – as a rule in the setting of an initial diagnosis - up to what extent a reversibility of PH exists depending on the extent of "remodeling" in the pulmonary vascular bed which, basically, is characterized by vascular wall thickening and increased collagen deposition /accumulation in the vascular wall, yet, as well, by thrombotic processes in microcirculation [2].

The non fixed component of PH, which is marked by vasoconstriction, can be acutely influenced by vasodilators, particularly those being highly selective for pulmonary vessels, resulting, in the best case, in a pressure decrease of pulmonary artery (PA) while Cardiac Output (CO) remains constant, to an increase of CO without essential changes in pressure, or to both parallel which all finds expression in a decrease of pulmonary vascular resistance (PVR). Pressure decrease that is not accompanied by an increase in CO or, at least, constant CO, is not regarded as positive. Three criteria are described for a positive vasoreagibility testing (VRT) for a defined main group of precapillary pulmonary hypertension (PAH, WHO group I: decrease of mean pressure of PA by at least 10 mm Hg, absolute decrease of mean pressure below 40 mm Hg and improved, or, at least, constant CO [3].

VRT is no standardized testing and is performed in a variety of ways at different sites. For instance, inhalable iloprost, inhalable NO, intravenous nitroprusside, intravenous milrinone, intravenous or sublingual Glycerol Trinitrate or sildenafil per os are used for the testing [4]. Testing per os is unfavorable for reason of is belated effect so that from experience and depending on the individually differing time of resorption, a waiting period of more than 60 minutes can become necessary to be able to record the maximal vasodilatory effect. Intravenous (I.V.) administration of sildenafil for therapy of PAH has been licensed/allowed since the beginning of 2011, namely if oral dosing temporarily is impossible, which opens up new perspectives for the performance of VRT as well.

To what extent positive reactions in the setting of the testing help predicting positive effects under constant therapy of all the above mentioned inhalable or orally applicable substances remains unclear. It is merely validated that patients with idiopathic PAH showing a positive reaction on VRT can be successfully treated with calcium antagonists [5, 6].

It is likewise uncertain whether the criteria defined for a successful testing for PAH are also valid for other forms such as PH in left heart failure (post capillary PH, WHO group II) [7, 8].

Objectives

The present study seeks to clarify the following questions as a pilot study:

1. Is sildenafil (Sil) administered intravenously effective, well tolerated and beneficial for vasoreactivity testing in precapillary PH?
2. Is efficacy comparable with established drugs such as inhalable iloprost or are there differing effects?
3. Is it possible to predict the clinical efficacy of sildenafil per os by testing it intravenously?
4.

Primary endpoint:

- Safety and efficacy of intravenous sildeanfil during vasoreactivity test in patients with precapillary pulmonary hypertension

Secondary endpoints:

- 6MWD
- RHC
- correlation of a positive test result in VRT according to the established criteria with clinical success of the therapy
- functional class
- NTproBNP

Study design

The trial is designed as mono-center, prospective, open label study (proof-of-concept-study) with historical control group.

Potential participants (patients with connective tissue disease or pulmonary primary disease, otherwise suspected pulmonary hypertension) are informed in detail about the study. A 24 hour time for consideration is followed by another discussion and, possibly, the patient's signature of the patient informed consent form. Afterwards, screening is performed via echocardiography and spiroergometry as well as the taking of blood samples (including NT-proBNP). Invasive diagnostics (right heart catheterization) has to follow after a maximum of 5 days in case there is an indication of pulmonary hypertension in order to verify the diagnosis of PH. In this setting, patients with manifest precapillary PH (PA pressure mean $\geq$ 25 mm Hg and pulmonary capillary wedge pressure PCWP $\leq$ 15 mm Hg) are selected by

means of pressure measurements at rest. Patients with a PCWP > 15 mm Hg can not participate in this study.

A complete diagnostics at rest and during sub maximal exercise (bicycle ergometry in supine position) is followed, after a resting period of 30 minutes, by the vasoreactivity testing with sildenafil citrate intravenously in all participating patients. The dosage is the approved dose of intravenous sildeanfil (10 mg intravenous sildenafil). Application of the I.V. preparation is to be performed as short infusion over a period of 10 minutes.

A comprehensive registration of hemodynamic and clinical changes at rest and under another sub maximal exercise testing has to follow. One day after, patients receive 20 mg sildenafil per os for the first time. Thirty to sixty minutes after the oral administration another spiroergometry is performed.

Patients are then all treated with 3 x 20 mg sildenafil per os over a period of 3 months followed by a control visit with all tests performed initially including right heart catheterization, yet, in particular, an echocardiographical evaluation of right heart function with regard to an improvement under therapy with sildenafil.

Trial Center:

- Kerckhoff-Klinik gGmbH Bad Nauheim (Bad Nauheim, Germany)

Diagnostic Treatments and Criteria

1. Comprehensive evaluation of hemodynamics is performed via a complete right heart catheterization with assessment of:
    - heart rate, blood pressure
    - pressure values:
        - right atrium (systolic/diastolic/mean pressure = s/d/m)
        - right ventricle (s/early diastolic/end diastolic)
        - pulmonary artery (s/d/m)
        - Pulmonary Capillary Wedge Pressure (PCW)
    - Cardiac Output (CO) and Cardiac Index (CI) via thermodilution and parallel according to Fick

- pulmonalery vascular resistance PVR, systemic vascular resistance SVR
- oxygen saturations: mixed venous (blood gas analysis), arterial (pulse oximetry)
- all parameters mentioned above except for CO according to Fick under bicycle ergometry

2. Complete transthoracic standard echocardiography.

   Right ventricular (RV) function is here to be considered in particular. An essential parameter of RV function is the so called TAPSE (Tricuspid Annular Plane Systolic Excursion) with a determined norm limit of 16 mm. Further echocardiographic right ventricular parameters such as end diastolic RV diameter, percental change of cross-section area (FAC), systolic peak velocity of lateral tricuspid annulus in tissue Doppler, extent of tricuspid insufficiency (TI) and size of right atrium are all part of the assessment [9].
3. Clinical Evaluation through standardized spiroergometry (CPX) and 6 minute walk distance (treadmill ergometry, 6 MWD) prior to and after therapy with sildenafil allowing a comparison with numerous trials on the effects of therapy with sildenafil in pulmonary arterial hypertension (PAH).
4. Determination of laboratory parameters beside NT-proBNP and high sensitive troponin (hsTNT) comprises base parameters of liver and renal function, of electrolyte metabolism as well as blood count and the inflammatory marker CRP. High sensitive biomarkers for renal function, as NGAL and cystatin C, are determined as well.

<u>Calculated amount of patients:</u>

For this pilot study a sample size of 10 patients is planned.

The historical control group consists of 10 pts. with conventional testing (iloprost) during the last 2 years in our center. Patients in the control group are matched with regards to age, sex, severity and category.

A pressure decrease in the pulmonary artery (PA) of 10 mmHg or more in testing is generally seen as a positive result. If the study drug would cause a fall of 3 mmHg more than Iloprost, this would be clinically significant.

Inclusion criteria

- Patients with connective tissue disease and manifest PH (PAH) according to the above criteria for PA pressure, but normal PCWP ($\leq$ 15 mm Hg at rest)

Exclusion criteria according to sildenafil contraindications

- Patient history of intolerance towards sildenafil
- Preexisting therapy with sildenafil
- Non arteriitic anterior ischemic optic neuropathy (NAION) as well as history of hereditary retinal diseases
- Severe hepatic cirrhosis (CHILD C)
- Renal insufficiency requiring dialysis treatment as well as strongly impaired renal function (GRF < 30 ml/min/m²)
- Stroke or myocardial infarction within the last six months
- Arterial hypotension (systolic blood pressure repeatedly < 90 mm Hg)
- Serious myocardial ischemia, valve defect requiring surgical intervention
    - Inability to understand and/or sign patient informed consent, missing consent

Study protocol

Baseline

According to clinical standards initial procedures are:

- Anamnesis, physical examination
- Laboratory values: NT-proBNP level, hsTNT, NGAL, cystatin C; base laboratory values (blood count, hepatic and renal values, electrolytes, CRP)
- Echocardiography
- Spiroergometry (if applicable treadmill ergometry, 6 MWD)
- Right heart catheterization with vasoreactivity testing

Follow up

After 3 months control of therapy success:

- Laboratory values: NT-proBNP level, hsTNT, NGAL, cystatin C; base laboratory values (blood count, hepatic and liver values, electrolytes, CRP)
- Echocardiography
- Spiroergometry, treadmill ergometrie (6 MWD)
- Right heart catheterization

Duration of study

Inclusion of first patient within second half year of 2012, inclusion of last patient 2013

Data acquisition

Clinical data are recorded in the patient documentation system and are secondarily assessed. Data evaluation is performed at the study center Kerckhoff-Klinik, Bad Nauheim, Germany. Patient informed consent signed by patient and informing physician is filed in a study binder.

Study diagram:

	**inclusion**	**3 month FU**
10 patients with precapillary PH (PAH)	RHC, VRT	RHC, VRT
	echo	echo
	Spiroergometry, 6MWT	Spiroergometry, 6MWT
	lab	lab

References

[1] Guidelines for the diagnosis and treatment of pulmonary hypertension; Galie´, N. et al; ***European Heart Journal (2009) 30, 2493–2537***

[2] Comprehensive Invasive and Noninvasive Approach to the Right Ventricle–Pulmonary Circulation Unit: State of the Art and Clinical and Research Implications. Champion, H., Michelakis, E. et al. ***Circulation. 2009;120:992-1007***
[3] Pulmonale Hypertonie 2010; Rosenkranz, S. et al.; ***Dtsch Med Wochenschr 2010; 135:S64-S66***
[4] Pulmonary Vasodilators. Siobal, M. ***Respiratory Care. July 2007 Vol. 52 No 7.***
[5] The effect of high doses of calcium-channel blockers on survival in primary pulmonary hypertension. Rich S, Kaufmann E, Levy PS. ***N Engl J Med 1992;327: 76–81***
[6] Long-term response to calcium channel blockers in idiopathic pulmonary arterial hypertension. Sitbon O, Humbert M, Jais X, Ioos V, Hamid AM, Provencher S, Garcia G, Parent F, Herve P, Simonneau G. ***Circulation 2005;111: 3105–3111.***
[7] Updated Clinical Classification of Pulmonary Hypertension. Simonneau, G**. *Am Coll Cardiol, 2009; 54:43-54***
[8] Heart Failure and Pulmonary Hypertension. Shin, J., Semigran, M. ***Heart Fail Clin. 2010 April ; 6(2): 215–222***
[9] Guidelines for the Echocardiographic Assessment of the Right Heart in Adults: A Report from the American Society of Echocardiography. Rudski, L. G. et al. ***J Am Soc Echocardiogr 2010;23:685-713***
