# Supplementary material for: Intravenous sildenafil acutely improves hemodynamic response to exercise in patients with connective tissue disease
Source: PLoS One. 2018 Sep 20;13(9):e0203947. doi: 10.1371/journal.pone.0203947 (PMC6147445; doi:10.1371/journal.pone.0203947)
Supplement: S3 Document — (DOC) [file pone.0203947.s012.doc]

**
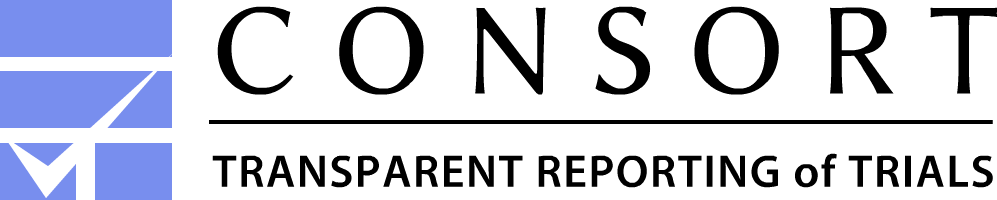
**

**CONSORT 2010 Flow Diagram**

**Allocation**

**Analysis**

**Follow-Up**

**Enrollment**

Assessed for eligibility (n = 21)

Excluded (n= 11)

♦  Not meeting inclusion criteria (n= 10)

♦  Declined to participate (n= 1)

♦  Other reasons (n= 0)

Analysed (n= 10)
♦ Excluded from analysis (give reasons) (n= 0)

Lost to follow-up (give reasons) (n= 0)

Discontinued intervention (give reasons) (n= 0)

Allocated to intervention (n= 10)

♦ Received allocated intervention (n= 10)

♦ Did not receive allocated intervention (give reasons) (n= 0)

No randomization
